# Irisin, a Link among Fatty Liver Disease, Physical Inactivity and Insulin Resistance

**DOI:** 10.3390/ijms151223163

**Published:** 2014-12-12

**Authors:** María Teresa Arias-Loste, Isidora Ranchal, Manuel Romero-Gómez, Javier Crespo

**Affiliations:** 1 Gastroenterology and Hepatology Department, Marqués de Valdecilla University Hospital, Santander 39008, Spain; E-Mail: digcgj@humv.es; 2 Infection, Immunity and Digestive Pathology Group, Marqués de Valdecilla Research Institute (IDIVAL), Santander 39008, Spain; 3 UCM Digestive Diseases and Ciberehd, Valme University Hospital, University of Seville, Sevilla 41014, Spain; E-Mails: isiranchal@yahoo.es (I.R.); mromerogomez@us.es (M.R.-G.)

**Keywords:** non-alcoholic fatty liver disease, insulin resistance, aerobic exercise, irisin, brown-fat-like development, muscle, FNDC5 (fibronectin type III domain-containing 5 transmembrane receptor), PPARγ (peroxisome proliferator-activated receptor γ), PGC-1α (peroxisome proliferator-activated receptor γ coactivator-1α)

## Abstract

Nonalcoholic fatty liver disease (NAFLD) is the most common cause of chronic liver disease in industrialized countries. The increasing prevalence of NAFLD mirrors the outbreak of obesity in western countries, highlighting the connection between these two conditions. Nevertheless, there is currently no specific pharmacotherapy for its treatment. Accepted management begins with weight loss and exercise. Moreover, exercise can provide metabolic benefits independently of weight loss. It is known how long-term aerobic training produces improvements in hepatic triglycerides, visceral adipose tissue and free fatty acids, even if there is no weight reduction. A recent study from Boström *et al.* unravels a potential molecular mechanism that may explain how exercise, independently of weight loss, can potentially improve metabolic parameters through a new messenger system (irisin) linking muscle and fat tissue. Irisin has been proposed to act as a hormone on subcutaneous white fat cells increasing energy expenditure by means of a program of brown-fat-like development. Moreover, it was also shown that irisin plasma concentration was higher in people who exercise, suggesting a molecular mechanism by which exercise may improve metabolism. The present systematic review is based on the possibility that irisin might represent a hypothetical connection between NAFLD pathogenesis and disease progression.

## 1. Introduction

The benefits of a balanced diet combined with exercise have been well documented, and they constitute the basis of the non-pharmacological treatment of cardiovascular and metabolic diseases [1]. In addition to improving resistance [2] and strength [3], physical exercise increases caloric expenditure, leading to a decrease in adipose tissue mass, and exerting important beneficial effects in the prevention of chronic diseases such as obesity and type 2 diabetes mellitus (T2DM) [4]. In patients with non-alcoholic fatty liver disease (NAFLD), the combination of physical exercise with decreased caloric intake is clearly beneficial; small reductions in body weight (between 6% and 10%) improve insulin sensitivity, decrease the necroinflammatory activity assessed by alanine aminotransferase (ALT), and improve lobular inflammation, ballooning, and steatosis [5,6]. Moreover, physical exercise can improve these parameters with no associated weight loss [7]. However, the mechanism or mechanisms by which physical exercise exerts this positive effect remain largely unknown. Identifying these mechanisms is an ongoing challenge in this field, which may lead to start exploring the development of drugs that could mimic these effects [8]. In this review, we aim to examine the current knowledge about irisin, a myokine that might represent a link between regular therapy and the clinical benefits in patients with NAFLD.

Brown adipose tissue can be detected in human adults by positron emission tomography (PET) [9,10], and its mass increases according to cold exposure [11] or physical activity [12]. The primary function of brown adipocytes is caloric expenditure, mainly related to raised uncoupling protein 1 (UCP1) expression. UCP1 causes an uncoupling in mitochondrial respiration promoting energy loss in the form of heat [13]. On the other hand, brown fat seems to be protective for metabolic diseases [14]. Two types of brown adipocytes have been described [15]: (1) Brown adipocytes that derive from myogenic mesenchymal cells and locate in the neck/supraclavicular, axillary and mediastinal regions and (2) “Beige” adipocytes found in the white fat. Being the later similar to white adipocytes, with a very low basal UCP1 expression, but able to respond, similarly to brown adipocytes, to cAMP-dependent increase in UCP1 expression and mitochondrial activity. Moreover, these beige adipocytes have a specific gene expression pattern—that enables distinguishing from white and brown adipocytes—that is controlled by physical exercise, and may cause a decrease in body weight and an improvement of glucose metabolism [16,17,18]. 

Physical exercise stimulates the production of myokines which are soluble factors released by skeletal muscle in response to muscle fiber contraction showing auto, para, and endocrine functions [19,20]. These myokines have been shown to participate as messengers among skeletal muscle, liver, adipose tissue, heart, brain, and blood vessels [21]. A large list of molecules have been recently considered as myokines, like angiopoietin-like 4 (ANGPTL4) [22], fibroblast growth factor-21 (FGF21) [23], interleukin-6 (IL-6) [24], IL-7 [25], IL-8, IL-15 [26], leukemia inhibitory factor (LIF) [27], myonectin [28], myostatin [29], vascular endothelial growth factor (VEGF) [30], brain-derived neurotrophic factor (BDNF) [27] and follistatin-like 1 (FSTL1) [31].

Physical exercise upregulates IL-6 [32], improving insulin sensitivity by increasing skeletal muscle glucose uptake and promoting fatty acid oxidation [33]. Nevertheless, plasma levels of IL-6 have been reported to be increased in obese patients [34] and IL-6 overexpressed in adipocytes from subjects with insulin resistance [35]. Moreover, it has been previously shown how an excess of IL-6 can also induce insulin resistance in hepatocytes [36], adipocytes, and skeletal muscle [37]. These data suggest that a strict balance is required to keep metabolism stable. Studies about skeletal muscle cells secretome in healthy and obese subjects, both at rest and in response to exercise, identified more than 1000 genes regulated by physical exercise [38]. 

Irisin, whose name derives from the Greek Goddess Iris (messenger of the gods) [39], is a recently described myokine whose levels seem to increase during physical exercise leading to heat generation and a possible protective effect on metabolic disorders [40]. Molecular mechanisms underlying Irisin, combined with the increase of brown fat, may unravel the basis of physical exercise benefits on different conditions. Irisin seems to induce a brown-like phenotype in some white adipocytes, which improves multiple metabolic parameters by increasing energy expenditure [41]. Therefore, irisin could play a hypothetical protective role against different conditions, such as cardiovascular diseases, type 2 diabetes mellitus (T2DM) or fatty liver disease. Moreover, through the improvement of obesity and its associated chronic inflammatory state, irisin may have a potential role in obesity-related cancer prevention as well as in osteoporosis and neurodegenerative diseases [42,43,44,45]. Irisin has emerged as a potential therapeutic target in metabolic diseases including non-alcoholic fatty liver disease (NAFLD), in which insulin resistance plays a major pathogenic role.

## 2. Mechanism of Action of Irisin

A few years ago, it was shown that peroxisome proliferator–activated receptor γ (PPARγ) coactivator-1α (PGC-1α)—a multispecific transcriptional coactivator, capable of regulating multiple genes in response to nutritional and physiological signals—is overexpressed in skeletal muscle after physical exercise [46]. This overexpression is associated with decreased body weight and lower levels of muscular inflammation markers and oxidative stress. It also improves insulin sensitivity by improving the efficiency of insulin signaling pathways. In order to elucidate how this cofactor mediate all these pleiotropic effects, Boström *et al.* [40,47] analyzed mice subjected to a chronic exercise program and observed that the overexpression of PGC-1α was associated with UCP1 upregulation in some adipocytes of subcutaneous fat. One of the factors potentially involved, fibronectin type III domain-containing 5 transmembrane receptor (FNDC5) [48], showed a clear increase in membrane expression. Moreover, it was shown how this receptor was cleaved, thereby releasing a molecule into the bloodstream, irisin, which may act remotely as a true muscle hormone. To test the function of FNDC5 *in vivo*, FNDC5 was isolated and inserted into adenoviral vectors, then injected into murine models of obesity. FNDC5 gene expression increased up to 15-fold, plasma irisin increased up to fourfold, and UCP1 expression increased 15-fold. Furthermore, these effects were accompanied by an increase in oxygen consumption, a decrease in weight, better glucose tolerance and reduced insulin secretion. Finally, a monoclonal antibody directed against irisin blocked the “browning” effects of exercise. 

Nevertheless, these data must be taken with caution according to recent publication of Raschke *et al.* [49]. This study analyzed genomic DNA, mRNA and expressed sequence tags and revealed that FNDC5, the gene encoding the precursor of irisin, displayed in humans a mutation in the conserved start codon ATG to ATA. HEK293 cells transfected with a human FNDC5 construct with ATA as start codon resulted in only 1% full-length protein compared to human FNDC5 with ATG. The authors suggest that data on mice may not be comparable.

Another aspect that was investigated by Boström *et al.* [50] was how UCP1 is upregulated, being the most likely mechanism the increased expression of PPARα. PPARα is a member of the family of PPAR ligand-activated receptors, which have roles in the control of lipid and glucose metabolism. FNDC5 overexpression increases the expression of PPAR mRNAs in white adipocytes up to 3-fold. In addition, the pharmacological inhibition of PPARα stops fat browning, suggesting a possible role of PPAR in mediating the effects of FNDC5 [51]. This relationship between irisin and PPARα signaling is extremely interesting, given that this signaling pathway is known to play a key role in hepatic β-oxidation [52].

**Figure 1:**
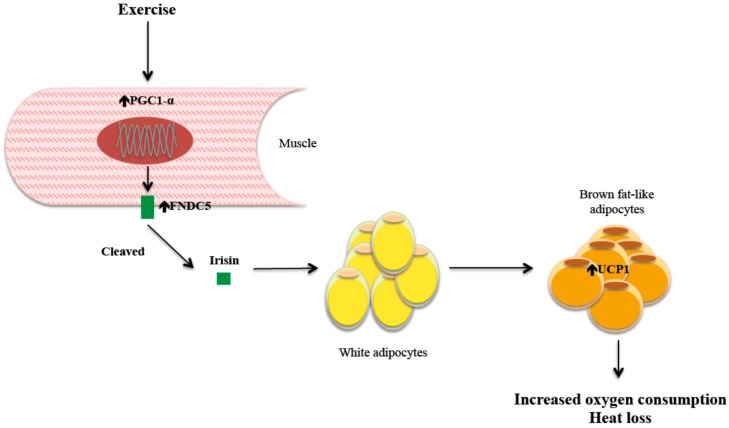
Irisin mechanism of action.

In summary, exercise seems to induce an increase of muscle PGC-1α, which is accompanied by greater FNDC5 membrane expression. FNDC5 is cleaved, releasing irisin, which goes into the general circulation and binds to a receptor or receptors (to be determined) on the surface of certain white fat adipocytes; and by a mechanism not yet fully elucidated (though in part involving overexpression of PPARα), UCP1 is upregulated, which is responsible for the resulting heat loss (Figure 1). 

Therefore, irisin apparently functions as a signal that communicates directly to muscle and adipose tissue, triggering a change in fat phenotype that becomes similar to brown adipose tissue [47]. This is accomplished through the activation of mitochondrial biogenesis and UCP1 expression, which lead to increased oxygen consumption and heat loss and therefore greater energy expenditure during exercise. Ultimately, this mechanism could be capable of activating a brown fat-like phenotype in white adipocytes [47], and it could be responsible for a better control of certain diseases related to insulin resistance [53], such as fatty liver disease. 

### 2.1. Irisin, Energy Expenditure, and Body Weight

Globally, obesity is associated with the chronic consumption of excess calories [54], although the energy requirement of each individual is highly variable. This variability has a huge impact on obesity, which is why clinical research on the potential involved genes is intense. A potential source of this variability is the amount of brown adipose tissue, and because irisin may induce the beige phenotype in some adipocytes, it could well be one of the determinants of the different energy needs. Plasma irisin level correlates with basal energy expenditure, although it is not directly related to changes in body weight [55]. Recently, an association has been shown between muscle expression of PGC-1α and FNDC5 and peak oxygen consumption in patients with heart failure undergoing an aerobic training program; the same was not seen in untrained patients [56]. Therefore, irisin could correlate with peak VO_2_, although plasma irisin was not measured. Leptin also induces white fat browning and increases thermogenesis and thus energy expenditure; this could explain the differences observed in subjects with similar irisin levels, although a relationship between energy expenditure and leptin has not been demonstrated [57]. The lack of correlation with leptin may be attributable to decreased responsiveness to leptin in obesity [58]. Although it is evident that many concepts need still to be defined, such as the maximum response to irisin or the existence of a potential irisin resistance, similar to what is being discussed for resistance to leptin, increasing irisin production can be a promising avenue for the prevention and treatment of obesity, and accordingly of NAFLD.

Plasma irisin level is related to some anthropometric parameters. The correlation between body weight or BMI and plasma irisin level has not been definitively established. Previous studies suggest a correlation between irisin and body mass index (BMI) [59,60]. A positive correlation with the diameter of the biceps has also been shown, which persists after adjustment for age, menopause, smoking, estrogen, and fat-free mass, suggesting that muscle mass is the main predictor of irisin production [61]. It is likely that these studies’ population type, sample size, and other methodological differences have had an impact on this discrepancy. However, it is possible that despite the upregulation of FNDC5 expression in the skeletal muscle of obese subjects, there would be no proteolytic FNDC5 cleavage responsible for the conversion of FNDC5 to irisin. In morbidly obese subjects, plasma irisin is low when normalized to body weight [62]. Considering that BMI normalization after bariatric surgery tends to promote metabolic normalization, an increase of irisin after surgery would be expected. However, Huh *et al.* [61] observed that plasma irisin and muscle FNDC5 were downregulated six months after surgical intervention (SI), which might reflect only the loss of muscle mass, although the mechanism underlying these effects cannot be determined and will require further studies. 

In any case, an irisin decrease does not seem to be responsible for the increase in energy expenditure or the normalization of insulin sensitivity observed after bariatric surgery [63,64,65]. In obese subjects who undergo bariatric surgery, plasma irisin seems to be higher in women than in men, suggesting that it may have different roles according to gender [61].

Similarly, in an experimental model of weight loss induced by caloric restriction, weight loss is not associated with changes in some proteins with distinct metabolic functions, such as BDNF, FGF21, IL-1β, myonectin, myostatin, and irisin [66]. These results cannot be considered definitive because of the possibility of a circadian pattern in the production of these proteins or a local action unquantifiable by analyzing serum. According to that study, a successful strategy to improve insulin sensitivity in subjects with NAFLD, obesity, and insulin resistance must address the increase in physical activity (with increased irisin and increased energy expenditure) and the fact that caloric restriction improves insulin sensitivity in other ways (for example, increasing adiponectin production) that are different from those of myokines.

### 2.2. Irisin and Exercise

Exercise comprises a series of actions that generate structural and metabolic changes in skeletal muscle, leading to increased caloric expenditure and decreased white adipose tissue mass. It also has important beneficial effects in the prevention of chronic diseases such as obesity and T2DM [4]. As previously shown, plasma irisin increase with exercise in both mice and humans. Moreover, its concentration correlates with the mRNA level of FNDC5, its precursor. According to previous studies, plasma irisin is higher in young individuals and increases clearly with acute anaerobic exercise, such as sprinting [61]. However, no significant irisin increase has been shown in human individuals undergoing chronic exercise training. This finding suggests that the exercise-induced decrease in muscle ATP results in the upregulation of irisin production, causing a short-term homeostatic effect. In this sense, some authors have only shown an irisin increase in very active elderly people [67]. If this hypothesis is confirmed, the role of irisin will not be very different from other myokines, such as IL-6, which increases immediately after exercising to facilitate heat loss but is not associated with long-term metabolic changes [21]. Therefore, current knowledge on the influence of exercise on irisin production is far from being fully understood, despite the fact that irisin has been postulated as an exercise hormone. 

## 3. Lipids and Irisin

Patients with fatty liver disease often have an atherogenic lipid profile characterized by a marked elevation of LDL and low HDL [68]. On the other hand, a key aspect in the pathogenesis of this disease is the existence of increased intrahepatic triglyceride deposits, with or without associated inflammatory and/or fibrotic phenomena. Intrahepatic triglyceride deposition is strongly correlated with BMI, waist circumference, blood pressure, insulin level, homeostasis model assessment (HOMA), and transaminase levels (alanine aminotransferase (ALT) and aspartate aminotransferase (AST)) [69]. As recently published by Zhang *et al.* [70], plasma irisin level seems to correlate negatively with intrahepatic triglyceride content, being significantly reduced in obese Chinese patients with NAFLD (without biopsy). A gradual reduction of irisin has been observed with increasing intrahepatic triglyceride content. The mechanism by which irisin prevents hepatic triglyceride accumulation could be direct or indirect. In this sense, irisin may modulate the PPARα signaling pathway, a key regulator of lipid metabolism that coordinates fat oxidation through a thermogenesis mechanism [51,71]. Furthermore, PPARα upregulates FGF21, which may lead to an improvement in hepatic steatosis and insulin sensitivity [72]. Therefore, irisin could regulate intrahepatic triglyceride content via FGF21. In addition, low plasma irisin is associated with increased ALT and AST, which suggests that irisin could behave as a protective factor against liver steatosis [70]. In patients with chronic renal failure, there is an inverse relationship between irisin and HDL [73]. HDL cholesterol protects against atherosclerosis by its inhibitory effect on cholesterol transport and anti-inflammatory effect [74]. HDL cholesterol is a clear predictor of vascular events in the overall population [75]. The inverse relationship between intrahepatic triglyceride level and irisin and the potential direct relationship between irisin and HDL reinforces the potential protective role of irisin, especially in patients with a chronic disease of high cardiovascular risk, such as fatty liver disease. These findings warrant further study of the potential role of irisin in the metabolism of HDL cholesterol [76]. In these same patients with chronic renal failure, plasma irisin correlates with insulin sensitivity. 

## 4. Irisin Plasma Level

Irisin concentration in serum can be determined by different commercialized enzyme-linked immunosorbent assay (ELISA), although the results obtained to date are conflicting and demand proper standardization. FNDC5 is undetectable in serum. As noted previously, irisin level is probably positively correlated with body weight, is higher after anaerobic exercise, and is different between men and women. Furthermore, a decrease of irisin with age has been suggested.

### Irisin Plasma Levels in Special Populations

In patients with chronic renal failure, plasma irisin is low [59]. Indoxyl sulfate decreases FNDC5 expression in cell cultures of skeletal muscle; this mechanism could explain the low plasma level of irisin in patients with chronic renal failure [73]. Another potential explanation could be related to the decrease in the muscle mass of patients with chronic renal failure. In one study, a questionnaire was used to analyze the quantity of physical exercise in the month before the study. In patients with noninsulin-dependent diabetes mellitus NIDDM, plasma irisin is lower than in controls without NIDDM [59,62,77]. A negative correlation has also been observed between HbA1c and plasma irisin [59]. On the other hand, irisin has been shown to be negatively correlated with HDL cholesterol and intrahepatic triglyceride content. Overall, irisin concentration may reflect in a comprehensive way the metabolic situation of patients with disorders related to metabolic syndrome. Irisinemia, a newly developed concept, could become a key tool in the management of some highly prevalent diseases, such as obesity, NIDDM, and proper fatty liver disease [78]. Although it is conceptually daring, irisinemia may constitute a surrogate marker for some of these diseases and could even be postulated as a therapeutic target [79]. 

## 5. Irisin’s Relationships with Adiponectin and Leptin

**Figure 2:**
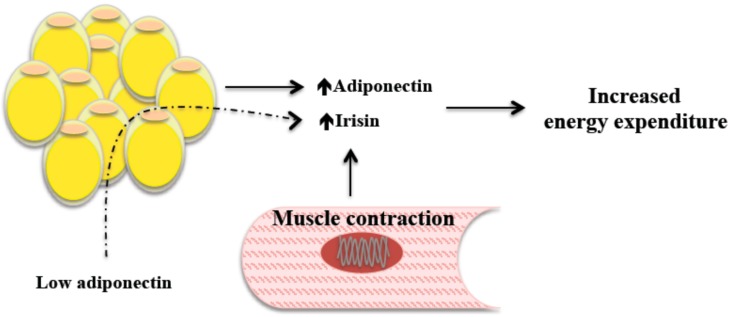
The elevation of irisin in response to low adiponectin can create a biofeedback loop to increase energy expenditure.

Adiponectin and irisin levels are negatively correlated. The elevation of irisin in response to low adiponectin can create a biofeedback loop to increase energy expenditure, as observed when FNDC5 injection decreased adiponectin concentration [40]. It is therefore possible that irisin exerts a direct effect on the adipocyte different from subcutaneous fat browning. Recently, the production and release of irisin by adipose tissue has been demonstrated, which may be an indication that irisin is an adipo-myokine and not just a myokine (Figure 2) [80]. 

Roberts *et al.* [81] identified a correlation between leptin level, total body fat, and FNDC5 mRNA levels in obese rats. These results are consistent with those of Timmons *et al.* [67] and Huh *et al.* [61]. In the first study, an interesting fact was also observed: PGC-1α, a regulator of FNDC5 gene expression, was upregulated in the triceps. Given that circulating leptin is increased in obese animals and that leptin can induce an increase of PGC-1α signaling [82], an increase in FNDC5 mRNA expression may be independent of physical exercise. In fact, a correlation has been observed between leptin plasma level and PGC1α and FNDC5 mRNA expression. Leptin may be a mediator between skeletal muscle and adipose tissue, forming a new compensatory mechanism that increases energy expenditure and fat loss via irisin. Furthermore, leptin treatment increases the expression of PGC-1α in skeletal muscle of *ob*/*ob* mice [83], although the potential interaction between leptin and irisin in energy regulation is unclear, particularly taking into account the leptin-resistant state. Both leptin and irisin activate signal transducer and activator of transcription 3 (STAT3) signaling, at least in the hippocampus; therefore, the concomitant effect of both is possible [44,84]. However, whether leptin is involved, there may be a yet unknown mediator released by adipose tissue that upregulates FNDC5 to induce heat loss.

Plasma irisin level is positively correlated with the concentrations of ghrelin and insulin-like growth factor-1 (IGF-1). Ghrelin stimulates growth and IGF-1 expression [85,86]. The potential interaction between irisin and other myokines is only known in a superficial way. A relationship between irisin and myostatin [87] has been observed, which may also improve insulin sensitivity [88]. The relationship between irisin and TNF-α is unknown [89].

## 6. Irisin and the Central Nervous System

Irisin has been detected in the brains of rodents and is capable of inducing neuronal proliferation at pharmacological doses. These findings suggest a potential role of irisin as a neurotransmitter, suggesting that the brain can effect some modulation on adipose tissue [44,45]. Other roles of irisin as neurotransmitter could explain the beneficial effects of exercise on neurodegenerative diseases such as Alzheimer’s disease [90,91]. 

## 7. Unresolved Issues and Future Research Avenues

The recent description of irisin involves significant uncertainty. In our opinion, these are the most important aspects to be investigated in the near future: 

Perhaps the most urgent aspect is the identification of the receptor or receptors that bind irisin. 

A hypothetical point at this time, but already suggested, refers to the potential tolerance or resistance to the action of irisin [55]. If any of these conditions were verified, they could explain the differences in the response to similar plasma levels of irisin. Knowing the mechanisms involved in irisin action would be even more important than the demonstration of tolerance or resistance. Alternatively, it could well be that irisin changes do not cause but result from a particular metabolic state, behaving instead as an adaptation mechanism to this state.

Determining and quantifying the induction of exercise-induced irisin and the type of exercise that can induce it. In this sense, it seems that acute exercise increases its release and chronic exercise causes its downregulation [61]. In other words, it seems that irisin is induced primarily by anaerobic exercise. In any case, determining the degree of benefit of physical exercise attributable to irisin [67] will be an endeavor of enormous complexity because more than 1000 genes are induced by exercise.

We must study the role of irisin in other diseases, particularly in diseases that may benefit from physical exercise and that are related to insulin resistance, such as NAFLD, cardiovascular diseases, and neurodegenerative diseases. 

## 8. Summary and Conclusions: Irisin and Non-Alcoholic Fatty Liver Disease

Physical inactivity leads to the accumulation of subcutaneous and visceral fat. For reasons not yet well known, a low-grade inflammatory process takes place in adipose tissue that contributes to the development of insulin resistance, facilitates atherosclerosis, promotes tumor growth, and induces neurodegenerative phenomena. In recent years, the spectrum of clinical manifestations associated with physical inactivity has been called the diseasome of physical inactivity. For many reasons, fatty liver disease may well be included in this diseasome of physical inactivity. Physical exercise is a key component of the change in lifestyle that is beneficial in NAFLD; in this context, a recent meta-analysis [92] showed that physical exercise is able to decrease hepatic steatosis even without associated weight loss. The mediator of this exercise-induced liver fat reduction is unknown, but the recent demonstration that plasma irisin level correlates negatively with intrahepatic triglyceride content suggests that irisin is involved in this result [70]. As we discussed previously, patients with NAFLD often have an atherogenic lipid profile. The inverse relationship between intrahepatic triglyceride level and irisin and the potential direct relationship between irisin and HDL reinforces the potential protective role of irisin, especially in patients with a chronic disease of high cardiovascular risk, such as fatty liver disease. 

In conclusion, irisin increases in response to acute exercise in both humans and mice in the following sequence: exercise stimulates the expression of PGC-1α, which promotes FNCD5 expression, eventually inducing an increase in plasma irisin. This causes the browning of white fat, increasing energy expenditure due to heat loss independently of exercise or food intake. Irisin causes an improvement of glucose homeostasis, insulin resistance, and obesity, mechanisms that are involved in the pathogenesis of NAFLD. It is essential that we improve our understanding of the complex interactions between muscle and other key agents of NAFLD; perhaps irisin is the main nexus between myocytes, adipocytes, and hepatocytes. In any case, the potential benefits of irisin are pending confirmation. It is likely that the beneficial effects of exercise cannot be provided by an “irisin tablet”, but it is equally true that a therapeutic intervention based on this myokine could be extremely useful in patients with difficulty performing conventional physical exercise. 

Although D. Santiago Ramón y Cajal stated that “only joy is a guarantee for health and longevity”, physical exercise could increase the coverage of this “guarantee”.

## Abbreviations

NAFLD: non-alcoholic fatty liver disease
UCP1: uncoupling protein 1
T2DM: type 2 diabetes mellitus
ATP: adenosin triphosphate
cAMP: Cyclic adenosine monophosphate
FGF21: fibroblast growth factor-21
interleukin-6: IL-6
TNF-α: tumour necrosis factor α
BDNF: brain-derived neurotrophic factor
CXCL-1: CXC motif ligand 1
PPARγ: peroxisome proliferator–activated receptor γ
PGC-1α: coactivator-1α
FNDC5: fibronectin type III domain-containing 5
BMI: body mass index
HOMA: homeostasis model assessment
NIDDM: noninsulin-dependent diabetes mellitus
STAT3: signal transducer activator of transcription 3
